# Detection of subgenome bias using an anchored syntenic approach in *Eleusine coracana* (finger millet)

**DOI:** 10.1186/s12864-021-07447-y

**Published:** 2021-03-12

**Authors:** Nathan D. Hall, Jinesh D. Patel, J. Scott McElroy, Leslie R. Goertzen

**Affiliations:** 1 Department of Crop, Soil and Environmental Science Auburn University, Auburn, AL USA; 2 Department of Biological Sciences, Auburn University, Auburn, AL USA

**Keywords:** *Eleusine indica*, Subgenome, Allotetraploid

## Abstract

**Background:**

Finger millet (*Eleusine coracana* 2n = 4x = 36) is a hardy, nutraceutical, climate change tolerant, orphan crop that is consumed throughout eastern Africa and India. Its genome has been sequenced multiple times, but A and B subgenomes could not be separated because no published genome for *E. indica* existed. The classification of A and B subgenomes is important for understanding the evolution of this crop and provide a means to improve current and future breeding programs.

**Results:**

We produced subgenome calls for 704 syntenic blocks and inferred A or B subgenomic identity for 59,377 genes 81% of the annotated genes. Phylogenetic analysis of a super matrix containing 455 genes shows high support for A and B divergence within the *Eleusine* genus. Synonymous substitution rates between A and B genes support A and B calls. The repetitive content on highly supported B contigs is higher than that on similar A contigs. Analysis of syntenic singletons showed evidence of biased fractionation showed a pattern of A genome dominance, with 61% A, 37% B and 1% unassigned, and was further supported by the pattern of loss observed among cyto-nuclear interacting genes.

**Conclusion:**

The evidence of individual gene calls within each syntenic block, provides a powerful tool for inference for subgenome classification. Our results show the utility of a draft genome in resolving A and B subgenomes calls, primarily it allows for the proper polarization of A and B syntenic blocks. There have been multiple calls for the use of phylogenetic inference in subgenome classification, our use of synteny is a practical application in a system that has only one parental genome available.

**Supplementary Information:**

The online version contains supplementary material available at 10.1186/s12864-021-07447-y.

## Background

*Eleusine coracana* (finger millet) is an important small-seed cereal crop in its native Africa and South Asia [1, 2]. It has been classified as a nutraceutical [3, 4] and has a panoply of uses from beer brewing to feed for livestock [5]. There are current efforts underway to improve the several landraces in both India and Africa, in addition to multiple genomics and transcriptomic projects [6]. These renewed efforts in this traditional and sometimes labor intensive crop [7] are driven by its under-developed economic potential and its ability to withstand the imminent abiotic stresses precipitated by climate change [6]. Understanding the origin of this crop will help a wide range of researchers improve the breeding efforts and understand the process of crop domestication. The crop plant, *E. coracana* is believed to be the product of an allopolyploid hybridization between *E. indica* and another likely extinct species [8–12]. *Eleusine indica* is consistently identified as an A genome donor [8, 9], however, based on the strength plastid phylogenetic analysis, some have suggested that *E. coracana* is the result of multiple hybridization events, between the B genome donor, *E. indica* and *E. tristachya* [11, 12].

The allopolyploid speciation event of *E. coracana* is potentially much older than most crop origin scenarios, occurring 1.4 Ma according to molecular clock estimates of plastid gene markers (*ndhA* intron, *ndhF*, *rps16*-*trnK*, *rps16* intron, *rps3*, and *rpl32*-*trnL*) [11]. It has been hypothesized that the original allopolyploidy was also the point of origin of the wild species *E. africana* (with an identical 2n = 4x = 36 chromosome number) that underwent domestication to form the crop species *E. coracana*, and evidence in support of this hypothesis is largely based on the phylogenetic analysis of small sets of single copy nuclear genes (e.g. *waxy*) [8, 11, 12], ITS and plastid markers [9, 13, 14], or cytogenetic methods [10]. To our knowledge, it has yet to be tested on a genomic scale.

Long considered an orphan crop [15], interest in *E. coracana* is gathering momentum [2]. Two genome projects [3, 16] have published results recently, including a scaffold-length assembly resolving many homeologs [16]. These assemblies provide foundational resources interpreting past studies investigating gene functions [17] and for understanding the origin and evolution of the A and B genomes. The key to unlocking these valuable resources is the genomic characterization of the most likely A genome donor *E. indica*, because it enables the separation of sub-genomes of a phased *E. coracana* assembly [16].

Assigning identity to phased polyploid assemblies is still time consuming and resource intensive even with access to advanced sequencing methods Single Molecular Real-Time sequencing [18], nanochannel genome mapping [19] and other approaches (e.g. HI-C [20]) which readily produce phased genomic assemblies [21]. Subgenomic phasing can be done with or without parental genomes. In the worst case scenario, homeologs are binned without a sequenced genome progenitor because it is extinct or unknown based on observed intrinsic differences between homeologous copies such as consistent biased fractionation caused by subgenomic dominance [22, 23], or differences in repeats [24, 25]. In cases where only one parental genome donor is known, the parental sequence is used to assign homeolog identity [8], relying on the assumption that the homeolog least similar to the parent is from the other parent. The optimal case where the genome donors are known, subgenomic regions or even transcripts are identified by their similarity to parents [26–28]. Genome painting has been widely employed with the use of probes and through in silico approaches analogs (e.g. the mapping of repetitive sequence from known progenitors to the allopolyploid [24].

The characterization of subgenomes is the first step in describing the subgenomic dominance that may occur when plants undergo diploidization and a single parental genome is preserved to a greater extent than would be expected by chance. It is marked by the smaller numbers of repetitive elements that it contains, and its preferential retention of single copy genes [29]. It occurs as the polyploid returns to diploid status following a whole genome duplication (WGD) event. Consistent patterns of subgenomic dominance and homeolog expression are conserved [30, 31]. Homeolog expression bias often occurs in tandem with subgenomic dominance but these two phenomena are not inextricably linked [32]. Homeolog expression bias may be precipitated by broad patterns of heterochromatin, more specific cases of methylation associated with transposable element (TE) clusters near down regulated genes, or novel interactions caused by trans-acting regulatory elements [31]. Down regulated genes may experience relaxed selection, undergo neofunctionalization [33, 34] and cause less of an impact if lost during the process of diploidization [22], or they may experience conserving selection in the presence of processes such as subfunctionalization [35, 36].

Subgenomic dominance may be driven by the effects of cytonuclear interacting genes.

Nuclear encoded cytoplasmic and organellar genes must coordinate expression and optimal macro-molecular structure in concert with each other and with organellar encoded genes to maintain normal function [37, 38]. Whole genomic duplication, events such as allopolyploidization, may perturb cytonuclear interaction and function due to addition of incongruous copy of nuclear genes. It is believed that a newly formed allopolyploid genome will attempt to retain the antecedent cytonuclear interaction of the maternal progenitor by suppressing the expression of the paternal cytonuclear genes [39]. These new patterns of expression that are the result of allopolyploidization are likely the basis for the selective advantage gained by polyploids [40].

Here we phase the most contiguous, publicly available genome to date [16], calculate synonymous substitution rate values to examine evolutionary relationship of the A and B genome to several members of the *Eleusine* genus and look for subgenomic bias across single copy cytoplasmic genes.

## Results

The A and B homeologs were identified using a strict syntenic approach. A and B calls were expanded using an in silico genome painting technique. All calls were checked against each other and using synonymous substitution rates to determine if A and B calls behaved as expected. Genome guided phylogeny was used to examine the relationship of A and B homeologs to closely related *Eleusine* species. Finally, we demonstrate evidence and examples of subgenome bias occurring within *Eleusine coracana*. The B subgenome shows a higher repeat content and higher total number of gene deletions within cytonuclear genes.

### Identification of a and B Homeologs employing Synteny

Synteny is the identification of homologous blocks of genes via conserved collinear patterns. The use of this method provides strong evidence for homology between genomes, but it provides limited coverage of the genomic assembly. To identify A and B genes a two step method was employed. First A and B calls were made using the direct gene to gene comparison. Gene relationships were determined using CoGe (Comparative Genomics) SynFinder to align the *E. coracana* genome to the *E. indica* genome with a syntenic depth of two to one respectively. This comparison created triads of genes, in which two *E. coracana* genes putatively from the A and B subgenome were linked by one *E. indica* gene. The *E. coracana* gene most similar to the *E. indica* gene was annotated as A while the other was annotated B. A total of 12,296 direct calls were made, 500 triads were uncalled because both *E. coracana* copies were equidistant from the *E. indica* copy. Direct calls were used to infer if the syntenic block they occurred in was A or B. If a block had significantly more A or B calls under chi square (*p* = 0.05), it was called A or B respectively; 704 blocks were called (351 A, 353 B) and 116 blocks had no call, 76% (88/116) of uncalled blocks contained fewer than 8 genes, in these cases 1 conflicting call was enough to keep the block from being characterized as A or B. Syntenic block calls were used to obtain contig calls. Contig calls were divided into three categories, strong, provisional and ambiguous. Strong contained no uncalled blocks. Provisional contained a single uncalled block, and ambiguous contained multiple uncalled blocks. A contig could be called as A, B or a crossover, if it contained both A and B calls. We identified 12 strong and 16 provisional crossovers, 68 strong and 29 provisional A contigs, and 82 strong and 23 provisional B contigs. The subgenome identity of 33% of the 62,347 predicted genes in *E. coracana* (9985 A genes and 10,349 B gene) was inferred usings strong syntenic region and surrounding sequence.

### In Silico genome painting

**Fig. 1:**
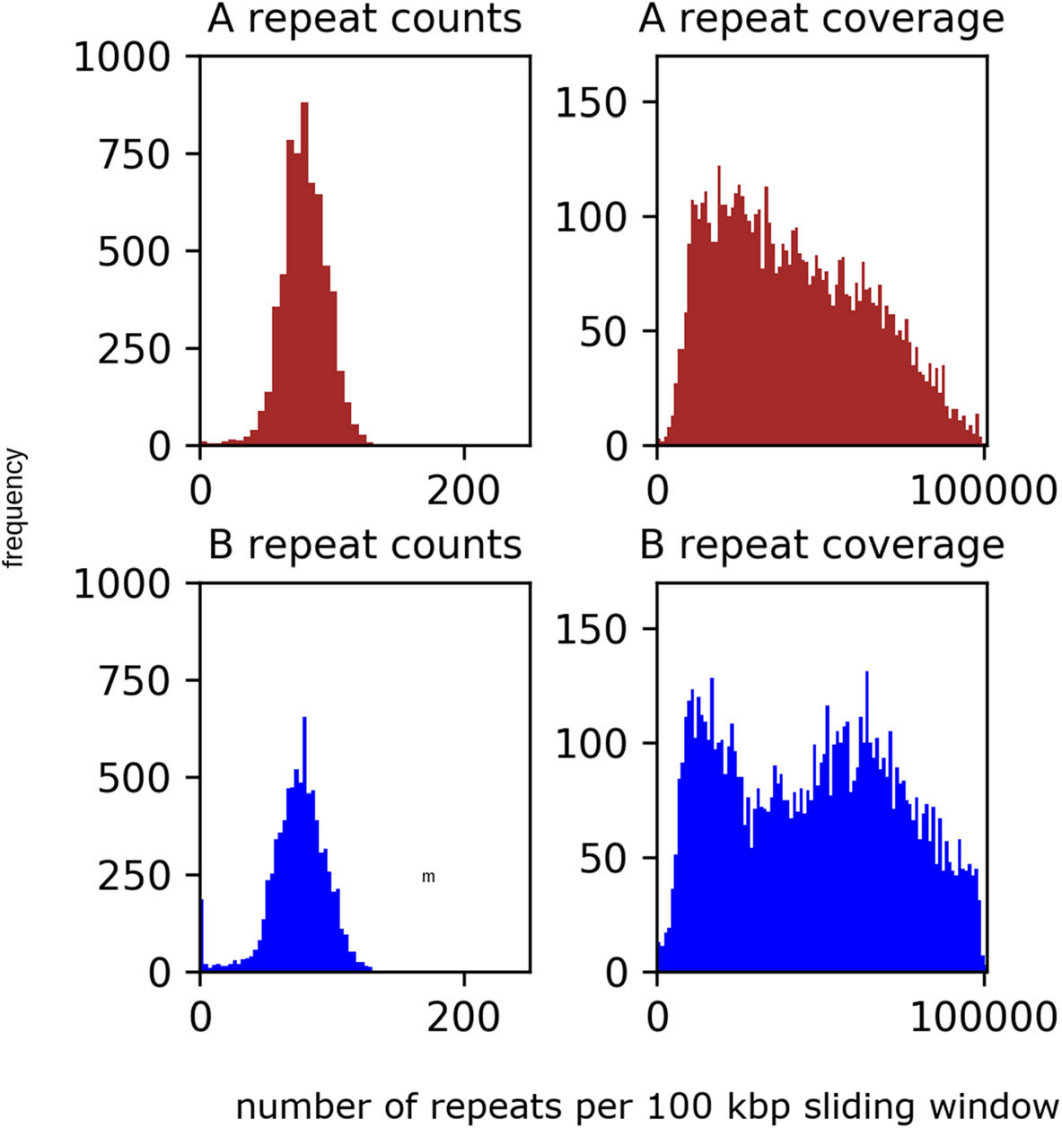
Histogram of the repetitive element coverage of 100 Kbp sliding window. Sliding windows from the A sub genome have a sum of 488,670 and a mean of 79.61 with standard deviation of std. 17.77, and B has a sum of 586,292 and a mean of 74.31 with a standard deviation of std. 22.25 number of windows 75% or greater coverage **a**:592 B:1375. **b** has a higher proportion of windows with 75% or greater coverage: **a** = 0.096, **b** = 0.174

A genome painting scheme using repetitive elements was employed to expand the number of A and B homeologs identified because a strictly syntenic approach limited the scope homeolog identification. Repetitive elements were identified for the entire *E. coracana* genome using RepeatScout with default settings and its output was used to create a custom database for RepeatMasker for annotation. A subset of A contigs (61) and B contigs (73) were chosen to identify A and B repeat elements. The B genome had a higher density of repetitive elements per sliding 100 kbp window (Fig. 1). A total of 50,416, repetitive elements longer than 200 bp were identified, 21,481 A and 28,935 B, spanning 81 Mbp (A 32 Mbp and B 49 Mbp). The longest repetitive elements spanned several thousand base pairs (A 12,578 bp) and (B 15,440 bp). For all retro-element families 898 were represented in both subgenomes by 84,965 entries (A 30,896 and B 54,069), while 180 families were uniquely predicted for one subgenome (A 50 and B 130) with 9281 entries (A 1175 and B 8106). Reads and their pairs that mapped to either A or B were extracted, and mapped against the entire genome. High quality coverage mapping was calculated for A and B read mappings where, both reads were mapped to the same contig and their insert was less than 1000 bp, with mapping score of greater than or equal to 30. A sliding window was used to sum all A and B reads mapped to a region of the genome, and region calls were made and aggregated using a custom python script (abPainting.ipynb). Using this method we called 31,543 A genes and 28,483 B genes. When added to existing cala total 59,377 unambiguous calls accounting for 81% of the gene annotations were made (Additional File 1).

### The identification of the a sub-genome donor

**Fig. 2:**
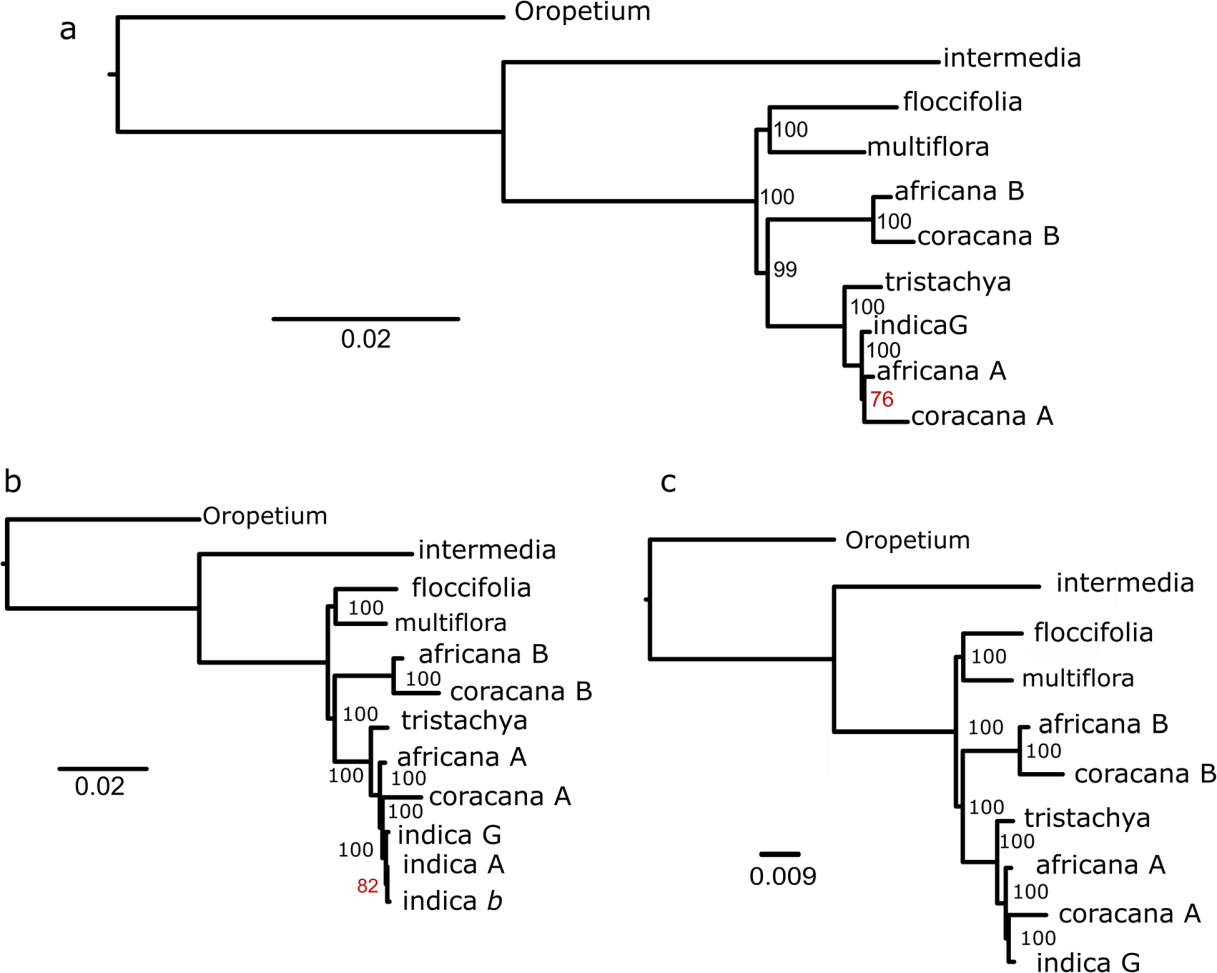
Backbone phylogenies produced using genome guided orthology approach and analysis done with RAxML, trees produced with figtree [41]. **a** 195 genes, a total length of 131,919 bp for 10 accessions. **b** gappy supermatrix that contains a putative B genes derived from *Eleusine indica* to test whether the targeted assembly method biases the assembled transcript to make them take on the characteristics of the genomic sequence used to capture the reads for assembly. It contains 31 false B genes derived from *E. indica.* Phylogenetic analysis confirms that they have not been biased by the underlying genomic sequence used to capture the reads for assembly. Gappy supermatrix contains 454 total genes and is 261,312 bp in length. **c** A gappy supermatrix which contains a 457 genes 277,536 bp in length

The A sub-genome donor was identified using a unified genomics approach, and the absence of the B sub-genome donor was identified in the largest set of species to date. We produced the largest *Eleusine* super-matrix compiled to date containing 455 genes to resolve A and B genome relationships within *Eleusine coracana* (Fig. 2abc). We tested the effects of targeted analysis on the *Eleusine indica* genome to determine if using a targeted assembly produced false B transcripts from a completely A genome. The results show that some, 31, putative B transcripts assembled, but that they were in the same clade as *E. indica* and the A genome (Fig. 2b). This demonstrates that the process of targeted assembly does not create B genomic transcripts as an artifact, or unduly bias the transcriptomic assembly process. The successful assembly and inclusion of 31 putative B transcripts suggests that our syntenic approach was not sensitive to cases of gene conversion, which would be expected when designating A and B genes by region. Phylogenomic analysis reveals that *E. indica* is indeed sister to the A genome and that *Eleusine tristachya* is sister to the *E. indica* - A genome clade while the B genome is sister the *E. tristachya* - A genome clade, further confirming that the B genome did not arise from *E. floccifolia* [10]. More complete species level sampling is required to determine the precise relationship of *E. indica* to the *Eleusine africana* A genome and the *E. coracana* A genome. According to a study by Zhang et al. [42] phylogenetic analysis supports *E. indica* as the maternal parent of *E. coracana* and *E. africana*, in addition to a close relationship between *E. indica* and *E. tristachya*, and between *E. floccifolia* and *E. multiflora*, and *E. intermedia* as a separate clade. So a rethinking of the labeling of ancestral genomes of *E. floccifolia*, *E. multiflora*, and *E. intermedia* maybe in order.

**Fig. 3:**
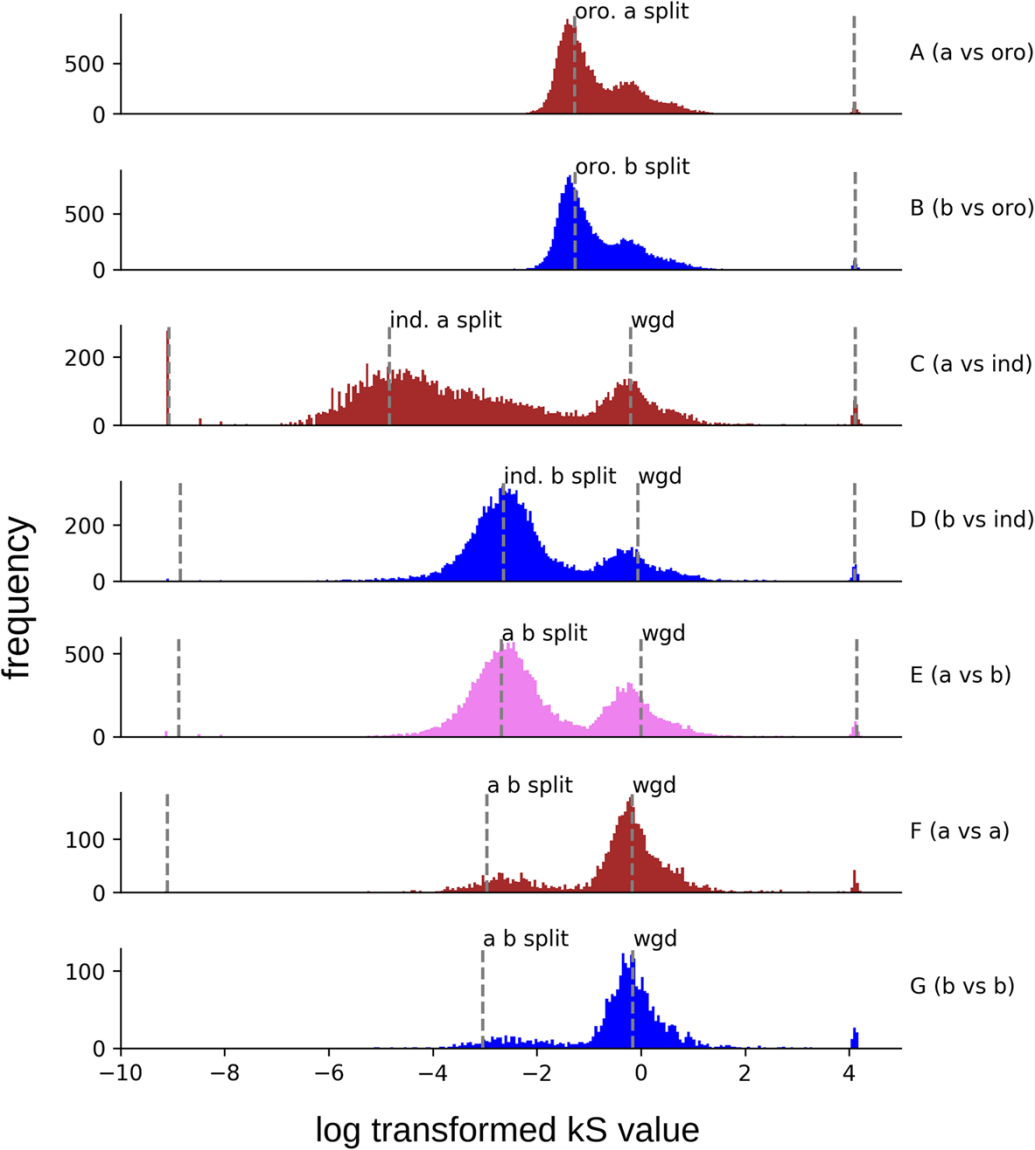
Histograms showing the distribution of synonymous substitution rates calculated using codeml in PAML, dashed lines were placed at local maxima detected using scipy.signal.find_peaks_cwt with a range of 10 to 15. **a** and **b** taken together clearly show the division between the A and B subgenomes of *Eleusine coracana* and *Oropetium thomaeum*. **a** 23,975 synonymous substitution rates between the A subgenome and *O. thomaeum* with local maxima found at − 1.27, and 4.1. **b** 20,834 synonymous substitution rates between the B subgenome and *O. thomaeum* with local maxima found at − 1.26, and 4.12. **c** shows the split between *Eleusine indica*. **c** 14,443 synonymous substitution rates between the A subgenome and *E. indica* with local maxima found at − 9.07,-4.84, − 0.201, and 4.12. **d** 13,923 synonymous substitution rates between the B subgenome and *E. indica* with local maxima found at − 8.85, − 2.64, − 0.063, and 4.11. **e** closely mirrors **d** because the A subgenome is closely related to *E. indica*. **e** 22,052 synonymous substitution rates between the A subgenome and the B subgenome with local maxima found at − 8.88, − 2.68, − 0.001, and 4.15. They also show the signature of the A and B subgenome split. A number of these likely represent gene conversions events that would have been invisible to our homeolog calling method and some number could be the result of error that can occur at regions of homeologous crossover leading to miscategorization at the extreme bounds of a crossover. **f** 4037 synonymous substitution rates between A and a with local maxima found at − 9.1, − 2.96, and − 0.173. **g** 2530 synonymous substitution rates between B and B with local maxima found at − 3.04, and − 0.16

Genome guided phylogenomic approach established an expected pattern of divergence among subgenomes for synonymous substitution rate analysis (Fig. 3). Synonymous substitution rate patterns confirm genome calls made by genome painting methods through the production of expected profiles between *E. indica* and A, and *E. indica* and B. The synonymous substitution rates suggest that there are a small number of mis-characterized genes or instances of gene conversion which would be expected given the size of the sliding window used to characterize A and B blocks. The comparison between *E. indica* and the A genome shows a peak at approximately 1.1 Ma when using the standard conversion rate of 6.5 e-9 substitutions per year [43].

### Analysis of repetitive content of the a and B sub-genomes

Analysis of repetitive DNA occurring on high confidence called contigs using RepeatMasker and custom repeat library indicated that the B region contains more repetitive elements per base pair than the A genome. Analysis of repeat family density within a sliding window of 100 Kbp was applied to A and B homeologs using syntenic blocks, and it revealed a significant difference in repeat count density for only one family, LTR_Copia, out of 30 families. Most families exist in clusters 1–20 per 100,000 bp, and DNA_Mule_MudDR is a striking example of a repeat occurring in dense clusters in the A genome, with a maximum of 114 in the sliding window, compared to 13 for the B genome. Several of the TE Line_L1 elements show an elevation in the density of single count insertions in the B genome which contained 1080 windows containing a single Line_L1 compared to the A genome which had 823 windows containing a single Line_L1 (Additional File 2). When all repeat counts and coverage are taken together they show that B contains more variation in repeat counts per sliding window while it also shows higher coverage than the A subgenome (Fig. 1). The B contigs had coverage of 75% or greater for 17.4% of all sliding windows compared to 9.64% in A contigs. The imbalance of repetitive content between subgenomes is also observed in other grasses such as *Eragrostis tef* [44]. This wider coverage of repeats is likely to have impacts on expression levels because of TE induced methylation (Fig. 1).

### B biased loss of Cytonuclear interacting genes

When the patterns of homeolog loss were examined at a global scale a strong pattern of biased fractionation favoring A genome homeolog retention was detected. A comparison of syntenic dyads created between hard masked assemblies of *Setaria italica* and *E. coracana* using CoGe, show that 61% singletons are A, that 38% are B, and 1% are unassigned (A: 1506, B:925, Unassigned: 21) (Additional Files 3,4,5).

To determine if cytonuclear genes showed appreciable subgenomic bias in their retention we started with a list of 4042 genes that were determined to be lost from either the A or B subgenome during an unmasked syntenic analysis. These genes occur in *E. indica* to *E. coracana* dyads not the expected *E. coracana* to *E. indica* to *E. coracana* triads. In the initial blast, 111 single copy genes that likely have potential cytonuclear interaction were identified. These were further examined in the *E. coracana* genome using CoGe (https://genomevolution.org/coge/ last accessed: 3/18/2020) blast which found that 26 of them had a single hit while eight of them had two hits but the second hit had either partial or poor alignment. The remaining 77 genes had either two or more hits. Out of the 34 genes, 24 were on the A subgenome and 10 were on the B subgenome (Additional File 6). These results suggest that genes on the A subgenome are favored for retention.

Thirty four genes were identified as single copy and involved several important pathways: defense signaling pathways, synthesis of indole-3-acetic acid, RNA interference, heat shock proteins, seed germination, plant growth formation and repair of photosystem II super complex, protein kinase, and flowering time. Some of the interesting genes that are important for normal functioning of chloroplast and mitochondria included *Met1,* Ribose-5-phosphate isomerase 3 (*RPI3*), Brassinazole insensitive pale gene-2 (*BPG2*) 3-hydroxyisobutyrate dehydrogenase (*HIBADH*), Translocase of outer membrane 34 kDa (*TOM34*).

## Discussion

Our findings for the A and B genome donors concur with past research [10, 13, 14, 45, 46]. The synonymous substitution rates show evidence of ancient Poaceae genome duplications [47–49], and suggested that when *Eleusine coracana* arose 1.1 Ma at the divergence of *Eleusine indica* and the A subgenome around the same time as *Eragrostis tef* [25]. Our synonymous substitution rates are within the range of previous predictions 0.50–2.7 Ma, albeit slightly more recent than the 1.40 Ma previously predicted [11].

Past work designating the genome donors of *E. coracana* has been limited to a few loci [8, 11, 12], or organellar genomes [46]. Our implementation of a genome guided orthology approach [50] is the most comprehensive treatment of this genus to date and the first to include a sample for each likely B genome donor since *Eleusine multiflora* and *Eleusine jaegeri* can be ruled out as genome donors based on the number of chromosomes alone and *Eleusine semisterilis* has never been considered a strong contender owing to its morphological divergence [51]. The placement of *Eleusine tristachya* sister to *Eleusine indica* - A genome (Fig. 2abc) clade is interesting because it opens the possibility that *E. tristachya*, the sole species of new world origin [52], was the result of an ancient long distance dispersal event. Previous work using plastid markers suggested that *E. tristachya* was only recently transferred to the new world [11]. The A genome - *E. indica* clade shows low resolution for the relationship among *E. indica*, *E. coracana* and *E. africana* (Fig. 2abc) in the most complete super-matrices calling into question the high bootstrap values in the gappy super-matrix (Fig. 2b). Here it appears that there is a positively misleading bias since the data rich genome based datasets are drawn together in the gappy super-matrix, while they are split in the ungapped super matrix.

*Eleusine coracana* still maintains several pairs of copy resistant genes as shown through BUSCO analysis [16]. Yet, a discernable genome wide bias toward A genome retention was detected in our syntenic dyad analysis, with 61% of the dyads determined to be A and 36% of the dyads determined to B. Furthermore, this pattern was upheld in the context of cytonuclear interacting genes where a total of 34 reversions of cytonuclear genes to single copy state heavily biased towards retention in the A subgenome. Four genes of note that were retained on the A genome are instrumental to growth, *Met1*, *RPI3, BPG2*, and *HIBADH*. *Met1* is a thylakoid-associated tetratricopeptide protein which is highly conserved in photosynthetic eukaryotes and major player in formation and repair photosystem II complex [53]. When the white light intensity was fluctuated, two independent *Met1* mutants showed reduction in growth, diameter of rosettes, biomass and PSII compared to wild type [53]. *RPI3* catalyzes the reversible conversion of ribose-5-phosphate to ribulose 5-phosphate in the non-oxidative phase of the pathway and photosynthesis process [54]. A map based cloning identified point mutation in ribose 5-phosphate isomerase (*RPI*) gene to cause reduction in cellulose synthesis, radical swelling and reduced growth of roots [55]. A *RPI2* knockout mutant showed abnormalities in chloroplast structure and function, reduced starch in leaves, delayed flowering and untimely cell death [56]. *BPG2* is a phytochrome-regulated gene which encodes protein required for normal chloroplast biogenesis and greening process [57]. Mutation in this gene can curtail accumulation of chloroplast protein induced by brassinazole, carotenoid pigmentation in the plastids and expression of *rbcL* and *psbA* and inefficient photosystem II and altered photosystem I function [57, 58]. *HIBADH* encodes a mitochondrial enzyme which catalyzes reversible oxidation reaction of 3-hydroxyisobutyrate to methylmalonate semialdehyde in presence of NAD+ [59]. It is also involved in degradation of branched-chain amino acids. Knockdown of this gene has reduced degradation of valine and isoleucine [60]. It seems apparent that these subgenomic biases are instrumental in shaping phenotypes and future plasticity of *E. coracana* no matter the driving mechanism*.*

## Conclusion

In conclusion, we were able to assign more than 80% of the *Eleusine coracana* genome into a subgenomic fraction. We were also able to discern cases of constitutive homeolog preference within our selected pathway consistent with the biased fractionation observed in cytonuclear interacting genes as well as genome wide biased fractionation related to repetitive element content of both subgenomes. Subsequent to the allopolyploid origin of the *E. coracana* lineage, the TE rich B subgenome has experienced more frequent gene loss than the A subgenome.

The TE rich B subgenome exhibited more frequent gene loss than the A subgenome. These classifications will aid researchers in improved genomic assemblies of *E. coracana*. Finally, our analysis provides breeders with extra information to fine tune marker assisted selection in breeding. Viruel et al. [61] recently highlighted the need for breeding programs to make use of wild relatives for the improvement of crop lines. These A and B subgenomic classifications can be leveraged to assist in understanding the biological mechanisms underlying these valuable and often quantitative traits that will lead to further crop improvement. In closing, this is the first genome wide analysis that firmly places the A subgenome donor as *E. indica,* and establishes the likely extinction of the B subgenome donor and absolute absence of the B subgenome donor from the sampled data.

## Methods

*Eleusine indica* genome (NCBI Accession: QEPD01000000) [42], was compared to the *Eleusine coracana DDBJ* DRA Accession: DRA005897 [16] genome using SynMap CoGe [62, 63] with quota align [64] set to limit two *E. coracana* to one *E. indica*, minimum number of aligned gene pairs was at 5, Ka\Ks values were calculated CoGe version of codeml [65] with a maximum value of 3 and a minimum value of 0. A vs B genome calls were made from raw downloaded CoGe output using with dc_ks_bagofgenes.py (https://github.com/NDHall/coge_tools/blob/master/dc_tools/dc_ks_bagofgenes.py last accessed: 3/31/2020). This approach first categorized *E. coracana* genes by the identity of their matching syntenic *E. indica* gene. *Eleusine indica* genes which possess only two *E. coracana* genes were used for the rest of the analysis. Since *E. indica* is known to be the maternal genome donor [3, 16] the *E. coracana* gene with the highest sequence similarity to *E. indica*, is designated A and the other is designated B. Designations were made per syntenic block using ab_call.py (https://github.com/NDHall/coge_tools/blob/master/dc_tools/ab_call.py last accessed: 3/31/2020) (*p*-value set as default and cutoff value of 5). A vs B calls per block are compared with chi squared analysis. If *p*-value is less than 0.05 the block is called as either A or B, depending on the dominant gene call. Syntenic blocks are then categorized by scaffold which are then designated A, B or AB depending on region calls and assigned level of confidence based on presence or absence of uncalled syntenic blocks. Genes that only had one *E. coracana* hit to one *E. indica* hit were extracted as a list and manually searched against the *E. coracana* genome to confirm singleton status, and searched against other meso-allopolyploids, rice and peanut genome to determine if these genes frequently revert to one copy.

### Transcriptome assembly

RNA-Seq reads were downloaded from NCBI (Additional File 7) and converted to fastq format with fastq-dump v2.8.2 from Sratoolkit v2.8.2–1 (https://trace.ncbi.nlm.nih.gov/Traces/sra/sra.cgi?view=software last accessed: 3/31/2020) and cleaned using fastp v0.19.4 [66] with default settings. Cleaned reads from diploid species were assembled using Trinity v2.4.0 [67] (−-max_memory 102, −-CPU 40, −-trimmomatic, −-fullcleanup, −-verbose). To produce the A and B sequences for tetraploid *E. africana*, its reads were mapped to *E. coracana* reference with Tophat v2.1.1 [68] employing Bowtie 2 v2.2.9 [69] with default flags. Bedtools intersect v2.27.1 [70] was used to extract A and B regions identified by the bag of gene approach for targeted assembly carried out by Trinity (−-genome_guided_bam, −-genome_guided_max_intron 10,000, −-max_memory 102G, −-CPU 40). To test if this process was artificially creating a B genome *E. indica* transcriptomic reads were also assembled to reference and split into A and B. Protein sequences were predicted using the TransDecoder v3.0.1 [71] software with programs Transdecoder.LongOrfs and Transdecoder. Predict which were run with default flags. Predicted proteins were condensed into a set of unigenes using cd-hit v4.7 [72] with default flags.

### Phylogenomics

Genome guided orthology [50] was implemented with ggOrtho (https://github.com/NDHall/ggOrtho/tree/master/gg_ortho last accessed: 3/31/2020). A set of reference genes were extracted by a comparison between *E. indica* and *Oropetium thomaeum* [18] using ggGetSet.py. A vs B genes were classified using region calls per gene and added separately as either A or B. Transcriptomes were matched to each gene as per Washburn et al. [50] using ggGoAdd.py. Multifasta files were then filtered with ggSelectAlns.py to exclude any file that contained more than one sequence per species and exclude any file lacking more than one. Alignments were made with codon aware MACSE v2 [73] run with default flags, trimmed with gBlocks v0.91b default mode, sequences were organized using fasta_ghost.py (https://github.com/NDHall/pysam_tools/tree/master/fasta_ghost last accessed: 3/31/2020) and concatenated using FASconCat v1.0 [74]. Concatenated sequences were partitioned by gene using PartitionFinder v2.0 [75]. RAxML v8.2.9 [76] was run with output from PartitionFinder v2.0, using GTR Gamma 1000 bootstraps.

#### Inferring *Eleusine coracana* a and B subgenomes

To extend A and B homeolog calls we decided to employ an in silico genome painting approach. To accomplish this end we began by selecting repetitive regions identified with repeatmasker on a subset of the previously identified A contigs. We chose this approach so we could test the concordance between A and B calls made with painting method and those made with a syntenic method. These elements were extracted, labeled as A or B and added to a common reference fasta to which and all reads from NCBI SRA DRR095893 were mapped. Each mapped read and its pair were extracted and labeled as A or B. During this process read pairs that were split between A and B repetitive elements were excluded. A and B reads were then mapped to the entire *E. coracana* genome and bedtools was used to calculate A and B read coverage for a sliding window of 250,000 bp in size that advanced 2000 bp per step. A custom python script (abPainting.ipynb) was used to determine A vs B bed regions. Regions were designated A (Additional File 8), B (Additional File 9), low coverage or ambiguous then calls were compared among all paint, and syntenic called regions, and unambiguous regions were reported using bedtools. Resulting unambiguous A and B bed files were used to extract a list of A and B genes from gtf file using bedtools intersect (Additional Files 10,11). Call accuracy was confirmed on a set of test contigs excluded from the initial mapping.

### Synonymous substitution rate calculations

Modified DagChainer [77] files were downloaded from CoGe [62, 63] for *E. coracana* vs *E. coracana* and for *E. coracana* vs *Eleusine indica*, *E. coracana* vs *Oropetium thomaeum*. Gene relationships were extracted using dc2multiFasta.py (https://github.com/NDHall/ggOrtho/blob/master/util_scripts/dc2multiFasta.py last accessed: 3/31/2020). Sequences were aligned with MACSE, cleaned with Gblocks default settings. The synonymous substitution rates were calculated using codeml from PAML and annotated as A or B using unambiguous calls (Additional Files 12,13,14). PAML values were matched with KEGG pathway annotation and updated using a total unambiguous filtered list of A and B calls (labelAvsBKsKa.py).

### Singleton analysis

Here we first identified 4042 genes that are in single copy in the current *E. coracana* genome. These genes occur in *E. indica* to *E. coracana* dyads not the expected *E. coracana* to *E. indica* to *E. coracana* triads. We retrieved a list of genes from the *Arabidopsis* genome database in TAIR 10 (https://www.arabidopsis.org) which are directly or indirectly involved in cytonuclear interaction. These cytonuclear genes were identified with BLAST (basic local alignment search tool) with the single copy genes identified in the *E. coracana* genome sequence with e^− 10^. The single copy that had a match with the cytonuclear genes were searched with BLAST again in the *E. coracana* genome for a finalized single copy status and only single hit genes on the genome were selected to for functional annotation with blastx to UniProt (Universal Protein resource) database in NCBI (https://www.ncbi.nlm.nih.gov/ last accessed 3/31/2020).

To establish a global pattern genome bias we compared hardmasked *Setaria italica* (COGE ID: 12241) and *E. coracana* (COGE ID: 52747) genomes using COGE and syntenic depth of 1 to 2: This comparison created triads when both A and B copies were present and dyads when only the A or B copy was present. Syntenic linkages were parsed with basic command line tools and bedtools, homeolog identities (A,B, or unassigned) were carried out using regions called by our in silico genome painting process (Additional File 15).

## Supplementary Information

**Additional file 1: **Gene list and *Setaria italica* based annotation: A csv containing list of genes designated A or B along with functional annotation based on *Setaria italica*.

**Additional file 2:.** A comparison of TE abundance on A and B contigs: A pdf of TE abundance on the A and B subgenomic regions identified by syntenic comparison.

**Additional file 3: **A singleton list: List of syntenic singletons found on the A genome during a comparison of *Eleusine coracana* and *Setaria italica*.

**Additional file 4: **B singleton list: List of syntenic singletons found on the B genome during a comparison of *Eleusine coracana* and *Setaria italica*.

**Additional file 5: **Unassigned singleton list: List of syntenic singletons that could not be assigned to either genome during a comparison of *Eleusine coracana* and *Setaria italica*.

**Additional file 6:.** Cytonuclear singletons: Excel sheet an examination of syntenic singleton cytonuclear genes.

**Additional file 7:.** SRA accessions: csv of SRAs used in assembly and expression analysis.

**Additional file 8:.** Painted A calls: Bed file of A regions determined using in silico genome painting.

**Additional file 9:.** Painted B calls: Bed file of B regions determined using in silico genome painting.

**Additional file 10:.** Unambiguous A calls: List of all A genes that were called once or were concordant between syntenic calls and painting calls.

**Additional file 11:.** Unambiguous B calls: List of all B genes that were called once or were concordant between syntenic calls and painting calls.

**Additional file 12: **Codeml *Oropetium* results: Synonymous substitution rates from codeml results between *Eleusine coracana* and *Oropetium thomaeum*.

**Additional file 13: **Codeml *Eleusine coracana* results: Synonymous substitution rates from codeml results between *Eleusine coracana* and itself.

**Additional file 14: **Codeml *Eleusine indica* results: Synonymous substitution rates from codeml results between *Eleusine coracana* and *Eleusine indica*. (TSV 5344 kb)

**Additional file 15: **BASH commands: Bash commands and comments used to extract and count A and B syntenic singletons (dyads) for the *Eleusine coracana* to *Setaria italica* comparison.

## Data Availability

The data sets analyzed for this during the current the study are available in. NCBI (https://www.ncbi.nlm.nih.gov/), DDBJ (https://www.ddbj.nig.ac.jp/index-e.html), and CoGe (https://genomevolution.org/coge/) Genome IDs (*Eleusine coracana*:51576,*Eleusine indica*: 51674, *Setaria italica*: 12241).

## Abbreviations

BLAST: Basic local alignment search tool
CoGe: Comparative genomics
Ma: Million years ago
TE: Transposable element
WGD: Whole genome duplication
