# Supplementary figures and images for: Detection of subgenome bias using an anchored syntenic approach in Eleusine coracana (finger millet)
Source: BMC Genomics. 2021 Mar 12;22:175. doi: 10.1186/s12864-021-07447-y (PMC7953713; doi:10.1186/s12864-021-07447-y)

frequency

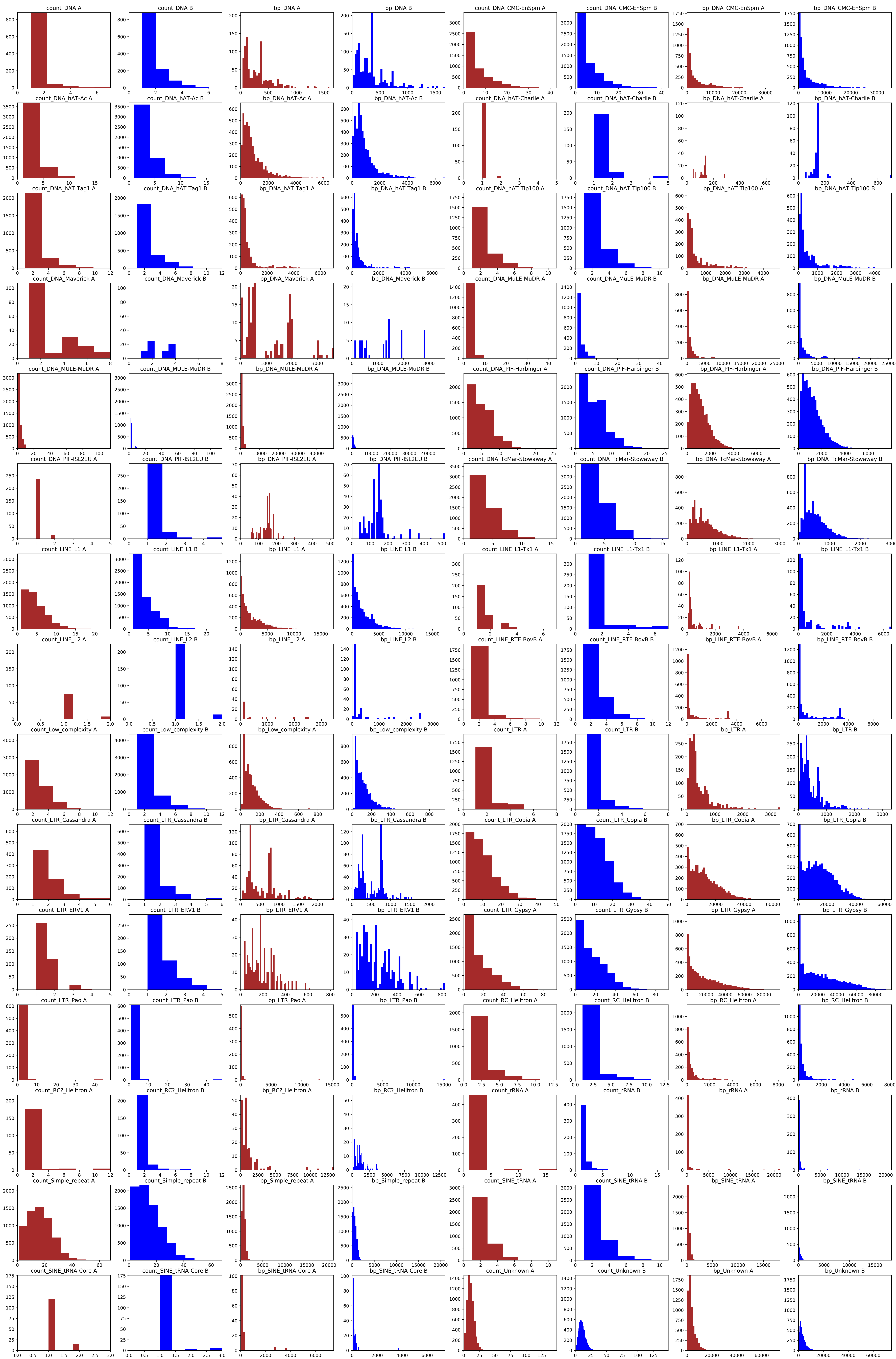

Number of repeats per 100 kbp sliding window

Supplement: Supplementary file 2 — Additional file 2:. A comparison of TE abundance on A and B contigs: A pdf of TE abundance on the A and B subgenomic regions identified by syntenic comparison. [file 12864_2021_7447_MOESM2_ESM.pdf]
